# Supplementary material for: Noise correlations and neuronal diversity may limit the utility of winner-take-all readout in a pop out visual search task
Source: PLoS Comput Biol. 2025 May 7;21(5):e1013092. doi: 10.1371/journal.pcbi.1013092 (PMC12088601; doi:10.1371/journal.pcbi.1013092)
Supplement: S1 Text — This file provides an analysis of the standard error of the mean (SEM) calculated over trials. It also presents (see S1 Fig in S1 text) the SEM, averaged over both trials and realizations, throughout the Results section. (DOCX) [file pcbi.1013092.s001.docx]

**Supporting information:**

$\boldsymbol{S}\boldsymbol{1}$ **- Standard error of the mean of the readout accuracy in simulations**

In this section, we present the standard error of the mean (SEM) of the readout accuracy, by exploring either SEM over trials or SEM over trials and realizations.

Standard error of the mean over trials: We conducted a series of 1000 Bernoulli trials, each yielding a binary outcome (success or failure). In statistical analysis, these trials serve as a random sample from a population characterized by a specific probability of success, denoted as $p$. For binary outcomes such as those in Bernoulli trials, the standard error of the mean is determined by:

$$SEM=\sqrt{\frac{p\left( 1-p \right)}{T}}$$

where $p$ represents the probability of success over trials, and $T$ denotes the number of trials. In our investigation, the estimated value of $p$ was represented by the readout accuracy along the y-axis. The precision of the readout accuracy was notably high, with the standard error of the mean limited to a very small magnitude that did not exceed $\frac{1}{2\sqrt{1000}}\approx0.016$.

For Figures $2D$, $2E$, $2H$, $2I$, $4A$, $4B$, $4D-4H$ we averaged the readout accuracy over both trials and realizations, the error is very small.

In Figures $S1.\{2D$, $2E$, $2H$, $2I$, $4D-4G\}$ the mean accuracy is depicted by the black line. To illustrate the variability, we plotted $mean\pm SEM$ in color. In Figures $S1.\left\{ 4A,4B, 4D \right\}, S2.\{5A,5B,5C\}$ the mean accuracy is depicted by the open circle. The error bars depict $mean\pm SEM$.

Note that in Figure $2A-2B$, and Figure $3A-3I, 3H, 3J$ there is no variability due to the neuronal heterogeneity. In figure $2A-2B$ the system is homogeneous. In Figure $3A-3I, 3H, 3J$ every point shows the result in a single realization of the neuronal heterogeneity. Hence, estimation error of the accuracy results only from the averaging over trials, but not over realizations; thus, the SEM is smaller than $0.016$.


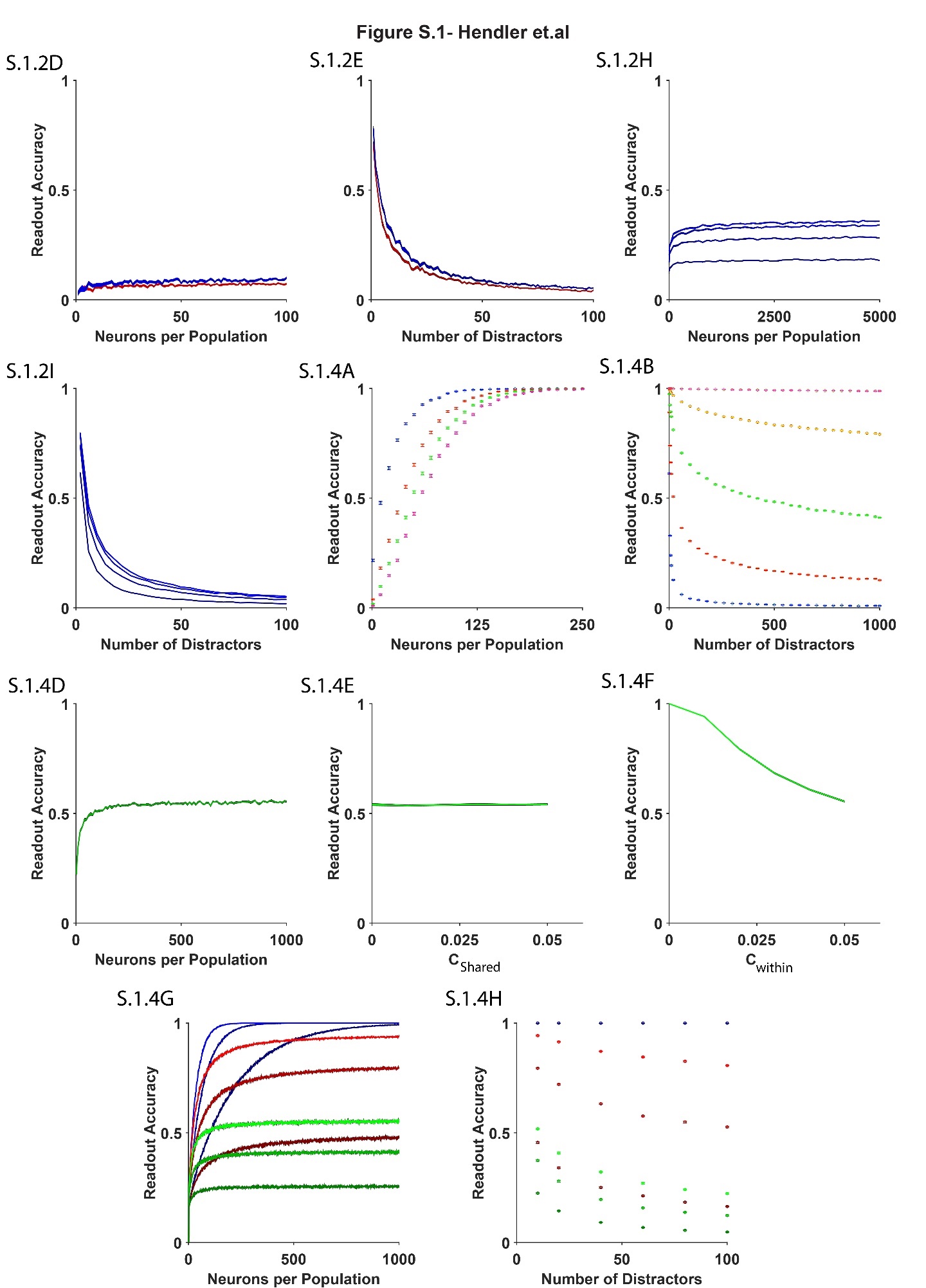


**S1 Fig | The error in estimating WTA and generalized WTA accuracy.** **(S.1.2D-E)** The accuracy of the single best cell WTA in heterogeneous systems is shown as a function of **(S.1.2D)** the number of neurons, $N$, and (**S.1.2E**) the number of distractors, $M$. The blue and red traces depict Poisson and exponential neuronal response distributions, respectively. **(S.1.2H-I)** WTA accuracy in artificial heterogeneous systems is shown as a function of **(S.1.2H)** the number of neurons, $N$, and **(S.1.2I)** the number of distractors, $M$, for different contextual modulation strengths, depicted by color. **(S.1.4A)** The accuracy of the generalized WTA is presented as a function of $N$. The different colors, depict different number of distractors, $M$. **(S.1.4B)** The accuracy is presented versus $M$. The different colors depict different values of $N$. Readout accuracy is presented in the cases of **(S.1.4D)** both $c_{\mathrm{shared}}\geq0$ and $c_{\mathrm{within}}\geq0$ correlations, **(S.1.4E)** $c_{within}=0.05,c_{\mathrm{shared}}\geq0$, **(S.1.4F)** $c_{\mathrm{within}}\geq0, c_{\mathrm{shared}}=0$. **(S.1.4G)** Accuracy as a function of the number of neurons per population, $N$. **(S.1.4H)** Accuracy as a function of the number of distractors, $M$, for large $N=10,000$. The colors in G-H represent different correlation levels and contextual modulation strengths. In Figures **S.1.(2D,2E,2H,2I,4D,4E,4F)** in order to illustrate the variability, we plotted mean±SEM in color. In Figures **S.1.(4A,4B,4D)** the mean accuracy is depicted by the open circle, and the error bars depict mean±SEM.
