# Supplementary material for: Noise correlations and neuronal diversity may limit the utility of winner-take-all readout in a pop out visual search task
Source: PLoS Comput Biol. 2025 May 7;21(5):e1013092. doi: 10.1371/journal.pcbi.1013092 (PMC12088601; doi:10.1371/journal.pcbi.1013092)
Supplement: S2 Text — This file shows (see S2 Fig in S2 text) the SEM of WTA and generalized WTA accuracy, as discussed in the Discussion section. (DOCX) [file pcbi.1013092.s002.docx]

**Supporting information:**

$\boldsymbol{S}\boldsymbol{2}$ **- Discussion section: The error in estimating WTA and generalized WTA accuracy**


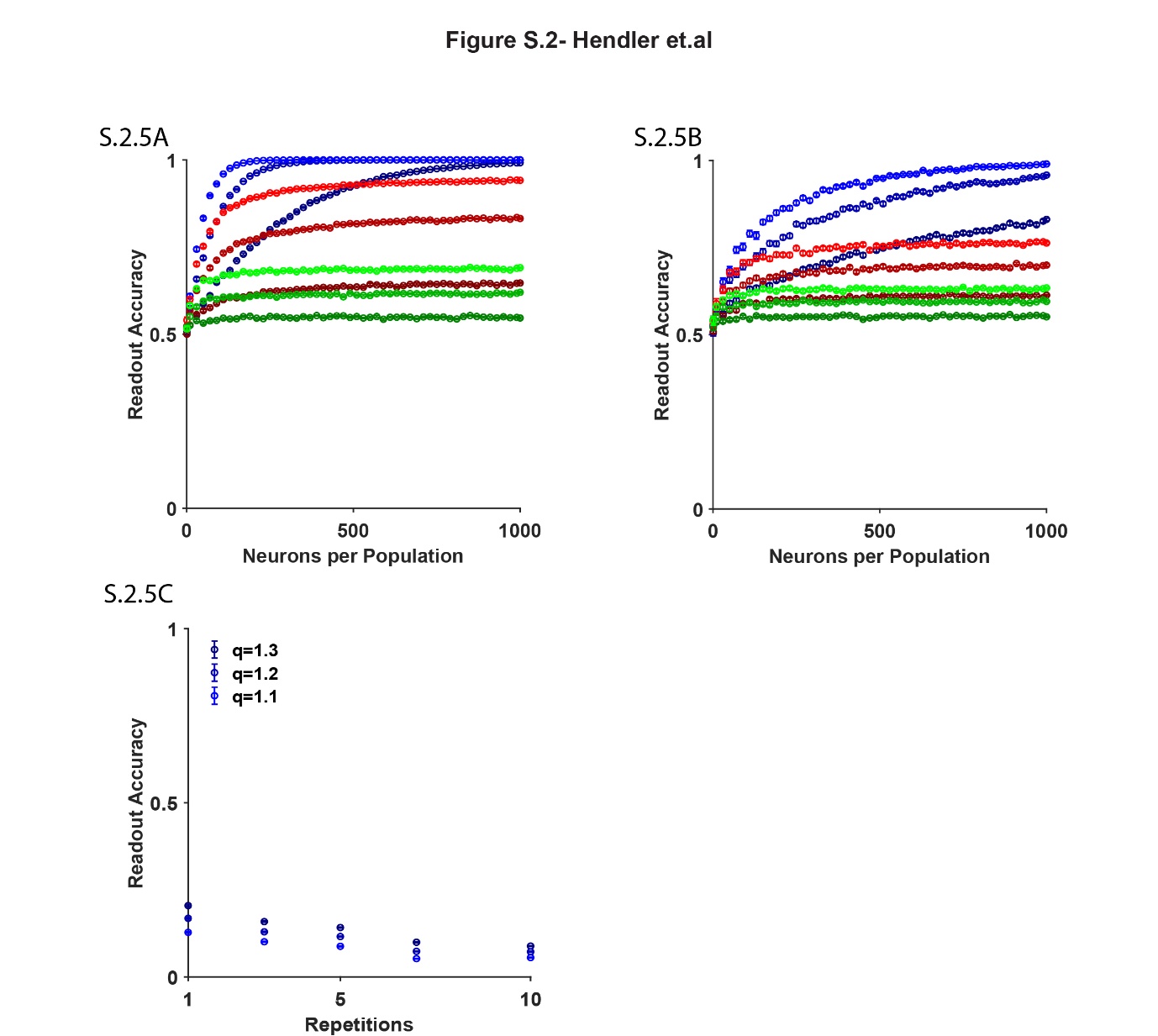


**S2 Fig | Discussion section: The error in estimating WTA and generalized WTA accuracy.** **S.2.(5A-B)** Readout accuracy for **(S.2.5A)** WTA, and **(S.2.5B)** generalized WTA, as a function of the number of neurons per population, $N$. Open circles and solid lines denote accuracy estimated via simulations and Equation $\backslash* MERGEFORMAT (21)$, respectively. Within-population correlations are indicated by different colors, and contextual modulation strengths by shades of these colors. **(S.2.5C)** Readout accuracy as a function of the number of repetitions, with different contextual modulation strengths depicted by different shades of blue.
