# Supplementary material for: Noise correlations and neuronal diversity may limit the utility of winner-take-all readout in a pop out visual search task
Source: PLoS Comput Biol. 2025 May 7;21(5):e1013092. doi: 10.1371/journal.pcbi.1013092 (PMC12088601; doi:10.1371/journal.pcbi.1013092)
Supplement: Attachment — Submitted filename: Response to reviewers.docx [file pcbi.1013092.s004.docx]

# Rebuttal Letter PCOMPBIOL-D-24-01308

The Winner-take-all readout mechanism fails to account for the high accuracy reported in pop out visual search

All reviewer comments are presented in black text, and our responses are shown in red. References to line numbers correspond to the clean version of the manuscript.

## Reviewer #1:

Visual search is a perceptual task in which a participant is instructed to locate an object of interest against a background of irrelevant objects. Of particular relevance to this manuscript is the finding that identifying a target amongst distractors that differ from the target by a unique visual feature is done automatically, with high speed and accuracy that are almost independent of the number of distractors. It is widely accepted that such “pop out” is achieved by winner-take-all (WTA) computation, and Hendler et al. use theoretical modeling to challenge this hypothesis. They convincingly show that heterogeneity in the neurons’ baseline firing rates critically limits the accuracy of such a model. The reason is that choices will be biased towards neurons whose baseline firing rate is relatively high.

The paper is interesting and insightful, and I have only few comments.

Relation to experiments:
The standard task in pop out tasks is to identify whether a unique object is presented. The answer to this question is yes/no and chance level is 50%. Indeed, in references (4) and (17), that are reproduced in Fig. 1D and 1E, respectively, these were the instructions of the participants. On the other hand, the question of the WTA algorithm is a different one: WHERE is the unique object? Hence chance level is reciprocal in the number of distractors. Therefore, using the latter to model the former is somewhat misleading. The authors should explain more precisely how their model can be mapped to the experimental results of references (4) and (17) or alternatively, discuss alternative experiments in which the task is to identify the location of the deviant.

Response: We thank the reviewer for highlighting the distinction between the localization [Harmening, W. M., Orlowski, J., Ben-Shahar, O., & Wagner, H. (2011)], [Ben-Tov, M., Donchin, O., Ben-Shahar, O., & Segev, R. (2015)] and detection [Treisman, A. M., & Gelade, G. (1980)], [Klein, R., & Farrell, M. (1989)] , [Müller, H. J., Heller, D., & Ziegler, J. (1995)] versions of the pop out task. We examined the detection task and found that its results are qualitatively consistent with those of the localization task. To address this, we have included new figures (see Figure $5A-B$) and new sections in the Methods [Lines $806-853$, $897-909$] delineating this point. We also address this issue in the Discussion [Lines $590-600$].

Related to this point, the authors highlight the difference between pop out, and the serial nature of search when the unique object is characterized by a unique conjunction of features (Figs. 1C and 1D). However, it is not clear how the difference between these two modes of visual search manifest in the model.

We now clarify in the Introduction that we focus "on the pop out search mode and in particular on its high accuracy" [Lines $90-91$]. Consequently, we do not address the conjunction of features in our model. While these questions are indeed important, our work emphasizes a gap in understanding that exists even at the level of a simple pop out task. We believe that addressing this foundational gap is essential before extending our analysis to more complex tasks.

A key component of the theory is the contextual modulation of the firing rate: the firing rate of a neuron to a stimulus presented in its receptive field depends also on the congruency of that stimulus with stimuli outside its receptive field. However, this is NOT what is presented in Fig. 1G, where we see the firing rate of a neuron when two different stimuli are presented to its receptive field (horizontal vs. vertical bar). This is certainly not what was done in reference 18 on which this figure is based. There, the stimulus in the receptive field was the same in the two conditions while the surround (context) varied. I guess that the illustration at the bottom of the figure is a typo. I suspect a similar typo related to Fig. 1H.

Corrected. We thank the reviewer for pointing this out.

Clarity:
It is strange that Figure 1I is only discussed in the Methods section.

We now discuss Figure $1I$ both at the very beginning of the Results [Lines $197-198$] and in the Methods [Lines $760-776$].

Line 79: the sentence “These studies… “ seems out of place

Corrected, we removed the sentence from the manuscript.

Lines 94-95: why is the time inherently independent of the number of distractors?

We have now improved the phrasing of the paragraph [Lines $119-124$].

Line 191: The notation “ceil” in N_ceil and p_ceil is confusing, given the fact that in Fig 2C p_ceil can be 0.7, far from ceiling. I suggest changing the notations

The reviewer is correct: this is indeed confusing. We have replaced the term "ceiling" with "critical” throughout the manuscript.

Fig. 2: What is the number of distractors in A? Also, x-label is missing from A and B
Line 237-239: what scatter plot (x)?

We now reference Table $1$, in the figure caption, which lists all parameter values [Lines $246-247$]. Additionally, we have edited the figures to ensure that the plot(x) is clear (cyan and magenta (x) markers), and the x-axis is properly labeled. We thank the reviewer for the careful and detailed feedback, which has greatly contributed to improving our manuscript.

## Reviewer #2:

This simulation study shows that two versions of the winner-takes all algorithm are unlikely to explain visual pop-out search. It makes an interesting point that is likely useful for future theorizing and empirical research; I could not discover any methodological flaws. From a cognitive perspective the basic idea seems a little naïve, though (but so does the highly successful salience model, this study is based on).
Signed. Heinrich Liesefeld

1. My only major critique of this study (which equally applies to the original Itti & Koch model, I believe) is that it is overly naïve from a cognitive perspective. Very early models of pop-out search already assumed that an initial attention allocation step that is informed by salience is followed by a verification step (Hoffman, 1979; see also Neisser, 1967 cited herein). The verification step is precisely introduced for the reason that basing a decision merely on the first (preattentive stage) is overly error prone. This basic assumption also made it into the currently most popular cognitive model of visual search (Guided Search, Wolfe, 1994, 2021). Another – somewhat less well known – solution to this problem is the repeated application of the same selection process (Humphreys & Müller, 1993, p. 59ff). I wonder how results would change if any of these slightly more sophisticated selection rules would be applied. For example, repeated application of the single-best-cell WTA would not suffer from the correlations in the generalized WTA and therefore yield similar results as the uncorrelated version (which made accurate predictions), I would think.

This is an interesting point: how would a more sophisticated algorithm perform? In the revised manuscript we report on how we tested the idea of repeated application of the WTA [Lines $677-693$, $925-940$ and Figure $5C$]. We found that for a Poisson population, repeated application of the WTA may be detrimental – under the constraint of a fixed total observation time. Nevertheless, other extensions of the WTA may improve the accuracy [Lines $695-719$]. Specifically, we mention: $1$) the use of neuronal heterogeneity. $2$) The use of the temporal structure of the response. In addition, we point out that our results are only valid under the assumptions of our model [Lines $720-743$]. Consequently, if noise correlations or the contextual modulation strength change with the number of distractors this may have a major impact on the accuracy of the WTA. We hope that this work will motivate the empirical community to study these features. These issues are now better clarified in the Discussion.

2. I am not sure I agree that divisive normalization can be relegated to future work. This is a highly influential idea in the context of salience/priority maps and if it had not been mentioned briefly in the Discussion (p. 29), I had brought it up in this review. This might just be my preference and for some other aspects, I would agree that these are beyond the scope of the present study, though. In general, some additional extensions (reducing the number of mechanisms that are “beyond the scope of the present study/work”) would certainly improve the potential impact of this paper.

Hoffman, J. E. (1979). A two-stage model of visual search. Perception & Psychophysics, 25(4), 319–327. <https://doi.org/10.3758/BF03198811>
Humphreys, G. W., & Muller, H. J. (1993). SEarch via Recursive Rejection (SERR): A connectionist model of visual search. Cognitive Psychology, 25(1), 43–110. <https://doi.org/10.1006/cogp.1993.1002>
Wolfe, J. M. (1994). Guided Search 2.0 A revised model of visual search. Psychonomic Bulletin & Review, 1(2), 202–238. <https://doi.org/10.3758/BF03200774>
Wolfe, J. M. (2021). Guided Search 6.0: An updated model of visual search. Psychonomic Bulletin & Review, 28, 1060–1092. <https://doi.org/10.3758/s13423-020-01859-9>

Divisive normalization is central to the field of saliency. Divisive normalization provides a mechanism that generates and amplifies the contextual modulation of the neural responses. In our work we focused on the accuracy of the WTA and not on the mechanism that generates contextual modulation. Consequently, we modeled the statistics of the neural responses following the empirical data, and did not model the mechanism that generates the neural response. This is now clarified [Lines $201-206$]. Also, we completely removed divisive normalization as a possible extension of our work.

In the revised manuscript we include a discussion of: $1$) WTA accuracy in target present- target absent tasks [Lines $590-600$ and Figure $5A-B$]. $2$) The accuracy of repeated application of the WTA. $3$) More sophisticated algorithms [Lines $695-719$]. $4$) We now better describe the directions we believe are the most interesting for future research [Lines $744-757$].

## Reviewer #3:

This is the review of "The winner-take-all readout mechanism fails to account for the high accuracy
reported in pop out visual search"

by Hendler, Segeve, Shamir, to Plos Computational Biology

This paper uses a modelling approach to argue that winner-take-all mechanisms cannot account for
the high accuracy in findinng out a pop-out item by humans and other animal species.
The model assumes a visual scene with a pop-out target item and many non-target items,
neurons respond to the target item and non-target items with different levels of
average responses, r_t and r_d, for the target and non-target respectively,
and their actual responses in a trial will be drawn from a probability distribution
whose averages will be r_t and r_d, with r_d = r_t/q by a q>1.
In an actual trial, the actual responses will not be the averages but
fluctuations around the averages, so that the highest response,
the winner response, from the population of neurons responding to the scene
may not be from a neuron responding to the target. Hence, the winner-take-all (WTA)
algorithm does not perform well compared to the actual performance of humans and
animals. The authors also considered an alternative, called
population-based WTA as opposed to single-best-cell WTA,
such that there are multiple neurons responding to each search item,
target or non-target, and the responses from the multiple neurons are averaged
to get an average response to each item, and the winner is then selected
from the winning average. They found that alternative also falls short.
In addition to pure modeling, the authors also used the neural data from archer
fish to give neural basis such as the value for q for their models.

The paper asks an interesting scientific question. However, there are
some serious problems with this paper that must be addressed.

(1) The paper aims to analyze the feasibility of the WTA readout by focusing on
issues such as number of distractors, population size, contextual modulation strength
(the value of q), heteogeneity of the neuronal population, and
noise correlations. In ths way, the paper builds various
probability distributions of the neural responses, and show
that WTA algorithm applied to the neural responses
could not account for the accuracies by human and animal data on pop-out tasks.
This conclusion is in contrast to that by Koene and Zhaoping 2007 and
Zhaoping and Zhe 2015. Using the WTA algorithm in the V1 Saliency Hypothesis,
and starting from human reaction times in pop-out search of some visual stimuli,
Zhaoping and colleagues quantitatively predict, with zero parameters, human reaction times
in pop-out search of some other visual stimuli, and these predictions match
observed data quantitatively.
The contrast between the conclusions of the current study and the previous works
suggest that this paper's models of probability distributions of the neural
responses (on which WTA must operation) may not be sufficiently
good models of the actual probability distribution of the neural responses.

Specifically, the probability distributions of the real
neural responses are most likely heavily influenced by recurrent interactions of
a neural circuit, such as the neural circuit in V1 (modeled by Li 1999,
also detailed in the textbook Zhaoping 2014), or the neural circuit in the optic
tectum of archer fish (see Zhaoping 2016 for a review of the evolution
of neural basis across species). The saliency mechanisms in these circuits
make neurons recurrently interact with each other via iso-feature suppression,
so that the neural responses to the pop-out target can be higher.
Such recurrent interactions in a neural circuit is likely to induce some kind of
fluctuations of the responses (for the target and non-target) that are not well
covered by the probability models in this paper. The introduction and discussion
of this paper should point out this limitation of the paper, and discuss
the relationship with previous works. Note that V1 as the neural basis for saliency
in primates has also strong neural data support, with simultaneous behavioral and neural
recordings from behaving monkeys doing a pop out task, by in Yan, Zhaoping, Li 2018.

This is a very important comment. We thank the reviewer for raising it. We now discuss the above studies. In the revised manuscript, we discuss the evidence for WTA in V1 in detail [Lines $667-676$] and make it clear that it deepens the puzzle [Lines $143-145$, $745-748$] - both in the Introduction and in the Discussion. Then, in the Discussion we expand on possible solutions that include: improved WTA algorithms [Lines $677-693$], incorporating the intricate feed-forward, feed-back and recurrent connectivity characteristics of the central nervous system [Lines $720-735$], utilization of the temporal aspect of the response [Lines $705-715$] or inherent heterogeneity [Lines $695-704$]. In addition, we discuss directions for future empirical research that are likely to impact WTA accuracy if taken into account [Lines $744-757$].

(2) It is very difficult to read and understand the content of this
paper, making it difficult to review the paper. There are two problems
in the writing. First, many of the scientific and logical arguments are delegated as
mathematical derivations to Method section rather than having them in
the main text. This is not appropriate, since these scientific and logical arguments
are the main (rather than supplementary) content of this paper, which is after all a computational
paper. Second, perhaps because they are in the Methods section, they are not written
in a comprehensible way, making it difficult for a reviewer
to judge scientifically whether the arguments are sound.
The most important content should be written in the main text,
in a way that is not only understandable by the reviewers, but also
understandable to general readers of the PLoS computational biology.
I suggest that the authors test the paper to naive readers (outside
their lab) to see whether the paper is comprehensible in scientific arguments and
technical details. I should be able to more appropriately review this paper after
a revision is made to address this readability problem.

Based on the reviewer's comments, we have edited and relocated significant portions of the Methods section to the Results section. This adjustment emphasizes the mathematical model, which is central to the paper, and aims to make it easier for potential readers to follow [Lines $170-200$, $211-242,$ $396-430$].

Below I also point out some other problems of this paper, some
to give specific examples where the writing is difficult
to read (I do not list all instances, just examples, so as
to give the authors an idea why they are difficult, to better
revise the paper), others to help with some important and
relevant references.

In the revised manuscript, we have edited parts of the introduction, results, and method sections to improve clarity and address the reviewer’s feedback. We hope that these changes make the manuscript clearer and more accessible, but we would be happy to consider further modifications if needed.

(3) Near line 100-103, references to Li 1999 PNAS should be added, it is
the first journal paper to explicitly propose a WTA algorithm on
V1 responses for saliency pop-out.

We have added the reference throughout the manuscript [Lines $124$, $668$, $732$].

(4) The introduction can be better organized. By line 118, I was
wondering why "The goal of the study report below was to examine whether WTA
consittitutes the mechanism underling the pop out in the visual system"
is a modeling question rather than an empirical question. Only later
did I see what the goal was. This can be improved.

We have added a paragraph to the Introduction outlining the aim of the study [Lines $143-156$].

(5) Figure 2 some plots do not have axes marked, difficult for readers.

We corrected Figure $2$.

(6) Equation (6), what is i, i', j, j'? The index i' appears in the
right side but not the left side of the equation.

Thank you, we corrected the equation [now labeled as Equation ($9$)] and explained it better [Lines $471-472$].

(7) The statement in line 484-486 has no reference!

We added a reference and edited the lines accordingly [now Lines $564-566$].

(8) The paper says that rate model neurons are used, is this clear
whether the probabilities distributions are for rate neurons
or spiking neurons?

Thank you for pointing this out. In our study we do not incorporate details of the temporal structure of the response, now clarified in the Results section [Lines $181-183$] and acknowledged as a limitation [Lines $721-724$, $733-735$].

(9) In line 667, Kullback-Leibler divergence --- divergence between what?

The Kullback-Leibler divergence measures how much the neuronal response distribution for the target stimulus differs from that for the distractor stimulus. This point is now clarified in the revised manuscript [Lines $768-771$].
