# Supplementary material for: Noise correlations and neuronal diversity may limit the utility of winner-take-all readout in a pop out visual search task
Source: PLoS Comput Biol. 2025 May 7;21(5):e1013092. doi: 10.1371/journal.pcbi.1013092 (PMC12088601; doi:10.1371/journal.pcbi.1013092)
Supplement: Attachment — Submitted filename: Response_to_Reviewers_auresp_2.docx [file pcbi.1013092.s005.docx]

# April $12$, $2025$

# Response to Reviewers PCOMPBIOL-D-24-01308R2

“Noise correlations and neuronal diversity may limit the utility of winner-take-all readout in a pop out visual search task”

All reviewer comments are presented in black text, and our responses are shown in red. References to line numbers correspond to the clean version of the manuscript.

## Reviewer #$1$:

The authors successfully addressed all my comments.

We thank the reviewer for the positive feedback.

## Reviewer #$2$:

Even though – as also pointed out by Reviewer 3 – the present approach does not reflect the state-of-the-art in cognitive psychology, I already found the previous version of the manuscript of value for theorizing on salience computations. From my perspective, it helps to justify the increased complexity of empirically-informed cognitive theories from a computational standpoint and some steps were taken in this direction. Overall, I am happy with the way the authors responded to the minor concerns from my previous review and can therefore recommend the manuscript for publication.

We thank the reviewer for providing a valuable perspective and helping us refine the scope of the manuscript.

## Reviewer #$3$:

This is the review of the revisiion of the paper "The winner-take-all readout mechanism fails to account for the high accuracy reported in pop out visual search"

I appreciate that much of the mathematical derivations of this computational paper
is now moved into the main text. I also get to understand the paper's content better
than I did in the original version.

We thank the reviewer for the encouragement and advice which enabled us to considerably improve the manuscript.

This paper uses a model of the visual responses x_i^j to a visual search item S_j,
and considers responses of many neurons to a visual search stimulus containing
a target t and many non-targets (or distractors ) d, and examines whether the
winner response ( i.e., the maximum x_i^j among responses with all possible i and j,)
is from a neuron responding to the target t in a pop-out search stimulus by a
sufficiently large probability (that reflects the high accuracy of human and animal's accuracy in
pop-out search). Hence, this model has two main components: (1) a model of the neural responses
to visual inputs, (2) a winner-take-all (WTA) algorithm to read out the winning response.

For (1), the paper assumes that x_i^j is a sample from a probability distribution
f(x_i^j |S_j), which is conditional to S_j (S_j = t or d), and this probability could be
conditionally independent between neurons x_i^j and x_{i'}^{j'}, or dependent on each other
by some correlation matrix C_{ii', jj'}.
The expectation value of x_i^j for S_t = t, i.e., E(x^j_i| S_j = t) and
that E(x^j_i| S_j = d) when S_j = d have a ratio q = E(x^j_i| S_j = t)/E(x^j_i| S_j = d).
The probability distribution f can be Poisson, exponential, etc. In addition,
the authors also used the Itti and Koch's saliency model to get the E(x^j_i| S_j = t) and
E(x^j_i| S_j = d) instead.

For (2), this study examined two WTA algorithms, one is the get the maximum response
among all x_i^j, the other is to average x_i^j across all i for a given j, and get
the winning average to identify the winning j.

The paper concludes on (2), the WTA algorithm, gives a poor accuracy to locate
the target. In other words, it often gives the winner, single neuron or average, as not one
responding to the target. This does not agree with the higher accuracy of pop-out search by humans and animals.

There is a main problem with this paper. The negative conclusion on (2), i.e., on WTA,
is conditioned on the model assumption on (1), i.e., on the model of the neural responses and their fluctuations.
The authors acknowledge this problem, in line 736-737, the paper states " ... the inherent tradeoff between
abstraction and biological realism emphasizes the need for caution in extrapolating our finding."
In particular, if (1) is grossly incorrect, then one should not attribute
the poor accuracy performance of the model to (2), i.e., to WTA. Hence, the title of the
paper is misleading as "The winner-take-all readout mechanism fails to account for the high
accuracy reported in pop out visual search". A reader seeing this title would assume
that WTA is the problem, when in fact the problem might be with the model of the
probability distributions of the neural responses to visual inputs. The authors
rightly pointed out that there are strong evidence for WTA in previous studies.
The title of this paper should be modified, perhaps to something like
"The winner-take-all readout mechanism fails to account for the high
accuracy reported in pop out visual search when neural responses to saliency are assumed as ... "

Corrected: We modified the title and edited the abstract [Lines $28-33$], in line with the reviewer's comments. We also added a few sentences before discussing points ($1$) and ($2$) to highlight these important ideas clearly [Lines $690-696$].

As pointed out in my last review, saliency neural responses are likely achieved by
a recurrent neural circuit, such as that in V1 in primates or optic tectum in archer fish
via the horizontal connections within a brain region. In particular, these horizontal
connections should make the response fluctuations in different neurons correlated
with each other by some kind of structure imposed by the recurrent interactions,
and they likely make the response to the target correlated with the response to
the distractors. Correlations in responses fluctuations could also arise from the arousal state of the animal.
These kinds of correlations are perhaps not according to the current model component (1) used by
the authors. Note that Itti and Koch's saliency model does not address this correlation in the
response fluctuations either, because that model does not use recurrent interactions to get the
responses to saliency. A biological plausible recurrent model for computing saliency is
Li (Zhaoping)'s model Li 1998 (which has all the model parameters listed,
the model parameters and many details are also available in the textbook Zhaoping 2014, if the authors
like to try it). That model uses noises in the neural dynamics (and noise induces response fluctuations),
but the saliency responses are robust and consistent with behavior. This paper should discuss this
issue as one of the main discussion points. To what degree the response fluctuations in a
recurrent neural circuit for saliency are related to the "Shared correlation" (shared in a particular way)
in this paper can be an interesting link for the discussion.

Corrected: We now discuss the important role of recurrent connectivity in shaping noise correlations and its effect on WTA accuracy [Lines $593-604$].

This paper does recognize this issue of
"correlation in response fluctuations", as it states in line 644- 647 " If future electrophysiological studies
provide new evidence that can challenge our basic assumptions on the statistics
of neural responses, this will greatly expand ...". However, not addressing this
issue more directly, when this issue looms so large, could undermine the contribution
of this paper, as this paper could be viewed as a contribution to point out that
simple models of response fluctuations (unlike that induced in a recurrent circuit)
fail to account for pop-out saliency.

Hence, one may say that the title of the paper
could also be revised to "Simple models of response fluctuations to saliency fails to account
for the high accuracy reported in pop out visual search when winner-take-all readout for saliency is used".
The authors could decide which title best highlights their contribution.

Corrected: We modified the title in line with the reviewer's comments. We also added a paragraph to the discussion highlighting the important role of recurrent connectivity in shaping noise correlations and its effect on WTA accuracy. We hope this study encourages and informs future modeling of noise correlations and key aspects of neuronal responses to pop out stimuli, discussed in [Lines $593-604$, $659-667$, $758-762$, $766-774$].

Additional comments:

There is still much room to improve the writing. E.g., in equation (2) , F_t, and F_d appear
without any definition. This is just an example, better writing could save reviewer/reader
a lot of time.

We took this comment to heart and made additional revisions to improve the writing. Note that $F_{t}$ and $F_{d}$ are now defined [Lines $182-184, 226$].

In the two sentences right after equation (9), maybe there is a typo, c1 and c2 should be swapped.

Corrected.

Line 479, neurons with closer tuning properties are more correlated --- this does
not necessarily mean that c1 >= c2. E.g., neurons tuned to the same orientations
can have very different receptive field locations.

Corrected: Here $c_{1}$ are for similarly tuned contextually modulated neurons with the same receptive field and $c_{2}$ for neurons with different RF (each responding to a different object). The confusion resulted from a typo right after equation ($9$), which has been corrected.

Line 628, "... what are often modelled by saliency maps [96-99]", please note that
the references 98 and 99 are not models of the saliency maps, but are experimental
evidence supporting the idea that the saliency map is in V1, using WTA readout.

We now cite this important evidence for WTA in the appropriate section [Line $143$].

Another strong piece of evidence, perhaps visually more direct, for the WTA is Zhaoping
and May 2007 (Psychophysical tests of
the hypothesis of a bottom-up saliency map in primary visual cortex ), see their Fig. 1,
it suggests that WTA is not done by the winner of the local population
average (like a summation model by Itti and Koch), but winners among single neurons!

Added [Line $143$].

In figures, hard to distinguish between curves of different color. Having figures at the
end of the paper (rather than with the text), away from the figure legends, without clear legends in the plots
to denote which curves are for what (so that I do not understand unless I find and read the figure legends
somewhere else), makes this paper time consuming for reviewers to
read and review. For example, Figure 2, A, B, D, E, what are thered, blue, and black curves for?

We thank the reviewer for the insightful comments and apologize for any inconvenience caused by these issues. We have improved figure clarity by adding labels directly within the plots, and the figures are now positioned directly above their captions for easier reference.
